# Unintentional injection of a dexamethasone implant into the crystalline lens: a case report

**DOI:** 10.5935/0004-2749.20200066

**Published:** 2020

**Authors:** Andrés Lisker Cervantes, Nicolás Crim, Santiago García-Arroyo, Virgilio Morales-Cantón, Raúl Velez Montoya

**Affiliations:** 1 Retina Department, Asociación para Evitar la Ceguera en México “Hospital Dr. Luis Sanchez Bulnes”, IAP, Mexico City, Mexico; 2 Cataract Department, Asociación para Evitar la Ceguera en México “Hospital Dr. Luis Sanchez Bulnes”, IAP, Mexico City, Mexico

**Keywords:** Retinal vein occlusion, Macular edema, Lens implantation, intraocular, Tomography, optical coherence, Dexamethasone/administration & dosage, Drug Implants, Phacoemulsification, Humans, Case reports, Oclusão da veia retiniana, Edema macular, Implante de lente intraocular, Tomografia de coerência óptica, Dexametasona/administração & dosage, Implantes de medicamento, Facoemulsificação, Humanos, Relatos de casos

## Abstract

The intravitreal dexamethasone implant is a sustained-release anti-inflammatory drug system that releases 0.7 mg of dexamethasone into the vitreous cavity. The following case report describes a rare complication: accidental injection of the dexamethasone implant into the crystalline lens. A 73-year-old woman was diagnosed with central retina vein occlusion and cystoid macular edema. Initial tSreatment included three monthly intravitreal doses of anti-vascular endothelial growth factor treatment, which was not successful. Treatment was then modified to an intravitreal dexamethasone implant. Ten weeks later, the implant was observed in the posterior cortex of the crystalline lens. Because no improvement had occurred, the patient underwent phacoemulsification surgery, during which part of the lens migrated into the vitreous cavity. Therefore, 23-gauge pars plana complete vitrectomy was performed with trans-surgical administration of intravitreal aflibercept. Crystalline lens injury due to an intravitreal dexamethasone implant is a rare complication and typically results from the injection procedure. Immediate surgical or conservative approaches should be considered on an individual basis.

## Abstract (pt)

O implante intravítreo de dexametasona é um sistema anti-inflamatório de liberação sustentada que libera 0,7 mg de dexametasona na cavidade vítrea. O relato de caso a seguir descreve uma complicação rara: injeção acidental do implante de Dexametasona no cristalino. Uma mulher de 73 anos foi diagnosticada com oclusão da veia central da retina e edema macular cistóide. O tratamento inicial incluiu três doses intravítreas mensais de tratamento com fator de crescimento endothelial anti-vascular, que não tiveram sucesso. O tratamento foi então mudado para um implante intravítreo de dexametasona. Dez semanas depois, o implante foi observado no córtex posterior do cristalino. Como não houve melhora, a paciente foi submetida à cirurgia de facoemulsificação, durante a qual parte do cristalino migrou para a cavidade vítrea. Portanto, foi realizada vitrectomia completa via *pars plana* 23-gauge com administração de aflibercepte intravítreo durante a cirurgia. Lesões no cristalino devido a implantes intravítreos de dexametasona são uma complicação rara e geralmente resulta do procedimento de injeção. Abordagens cirúrgicas ou conservadoras imediatas devem ser consideradas caso a caso.

## INTRODUCTION

The intravitreal dexamethasone implant (Ozurdex^®^; Allergan Inc., Irvine, CA, USA) is a potent sustained-release anti-inflammatory drug system that consists of a proprietary biodegradable copolymer matrix that releases 0.7 mg of dexamethasone into the vitreous cavity. The copolymer is a compound of lactic acid, glycolic acid, and micronized dexamethasone. The implant is designed to gradually release the total dose of anti-inflammatory drug over a 3- to 6-month period^(1)^; it is a preservative-free implant (6 mm in length and 0.46 mm in diameter) that is delivered into the vitreous cavity through a specially designed 22-gauge needle^(2)^. Its clinical use was approved by the Food and Drug Administration in 2009 for the treatment of macular edema secondary to retinal vein occlusion, noninfectious posterior uveitis, and diabetic macular edema^(3-5)^.

The intravitreal injection of drugs (including Ozurdex) has been associated with several adverse effects such as cataract formation, endophthalmitis, vitreous hemorrhage, hypotony, retinal detachment, and intraocular pressure (IOP) elevation^(6)^. Here, we describe a rare complication regarding injection of the implant.

## CASE REPORT

A 73-year-old woman presented to our clinic for treatment of central retinal vein occlusion and cystoid macular edema (CME). She had a past medical history of systemic arterial hypertension. Her best-corrected visual acuities were 20/30 in the right eye and hand motion in the left eye. Ophthalmic examination revealed a mild nuclear cataract in the left eye. Dilated fundus examination revealed severe tortuosity and engorgement of all branches of the central retinal vein, extensive deep blot and flame-shaped superficial hemorrhages, cotton-wool spots along the superior and inferior temporal arcades, and macular thickening in the left eye. Fundus examination in the right eye revealed normal findings.

**Figure 1:**
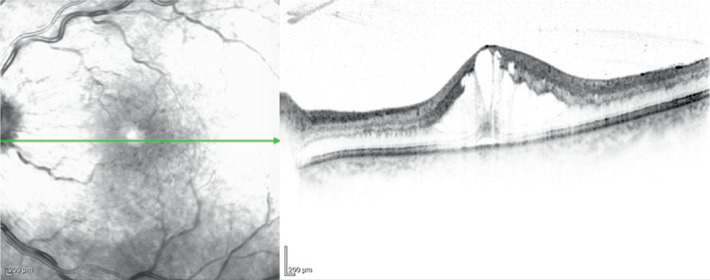
Optical coherence tomography of the left eye confirmed the diagnosis of cystoid macular edema.

Fluorescein angiography showed delayed arteriovenous transit, peripheral retina non-perfusion, hypofluorescence due to retinal hemorrhage, late staining of retinal veins, and optic nerve leakage. No retinal neo vascularization was present. Optical coherence tomography of the left eye confirmed the presence of CME (Figure 1). Initial treatment for CME included three monthly intravitreal doses of two anti-vascular endothelial growth factor drugs: two doses of bevacizumab (Avastin; Genentech, San Francisco, CA, USA) and one dose of aflibercept (Eylea; Bayer Healthcare Pharmaceuticals, Berlin, Germany); this treatment was not successful. The CME was determined to be unresponsive, and treatment was switched to an intravitreal de xamethasone implant (Ozurdex).

**Figure 2:**
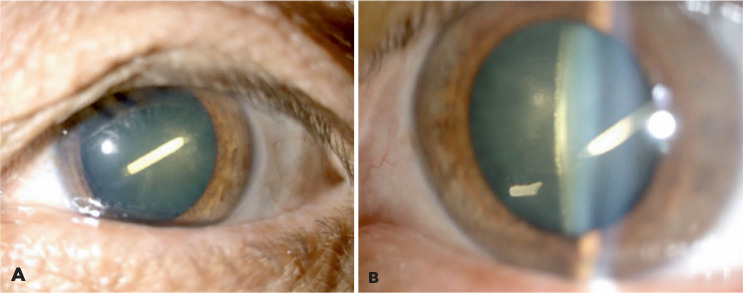
Slit lamp examination of the anterior chamber showed that the dexamethasone implant was lodged in the posterior cortex of the crystalline lens (A) and revealed a small fragment floating freely in the anterior vitreous, close to the posterior capsule rupture (B).

After Ozurdex injection, the patient was lost to follow-up. She returned 10 weeks later; in the left eye, her best-corrected visual acuity was counting fingers, and her IOP was 14 mmHg. Ophthalmic examination showed that the dexamethasone implant was lodged in the posterior cortex of the crystalline lens and revealed a small rupture of the posterior capsule (Figure 2A, B). The implant had fractured into two fragments; the smaller fragment was floating freely in the anterior vitreous, near the location of rupture in the posterior capsule. These findings were confirmed by ultrasound biomicroscopy. The anterior chamber was deep and showed no cellular reaction. Specular microscopy revealed a normal endothelial cell count (2592 cells/mm^3^). Left eye optical coherence tomography findings revealed a slight reduction in macular thickness. Because of the slight improvement of CME, combined with normal IOP and absences of corneal toxicity and anterior chamber inflammation, we chose a conservative approach without immediate surgical intervention, as well as close follow-up.

**Figure 3:**
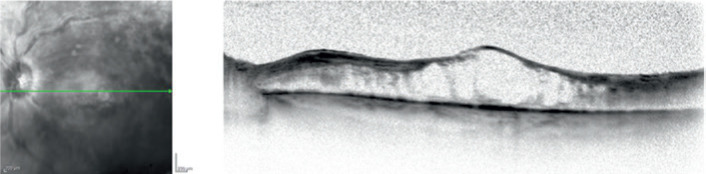
Left eye optical coherence tomography showed an increase in central macular thickness.

The patient returned 1 month later; she had a 3+ posterior subcapsular cataract, normal IOP, and normal corneal endothelial cell counts. Left eye optical coherence tomography showed an increase in central macular thickness (Figure 3). Because no further improvement was observed, the patient was offered phacoemulsification surgery and trans-surgical intravitreal aflibercept (15 weeks after the injection of Ozurdex). During the surgery, a portion of the lens migrated into the vitreous cavity. Therefore, 23-gauge pars plana vitrectomy was performed concurrently, without complications. After 2 months of follow-up with monthly injections of aflibercept, the best-corrected visual acuity in the patient’s left eye was 20/400, and marginal improvement of CME was observed.

## DISCUSSION

Treatments for ischemic central retinal vein occlusion with CME include panretinal photocoagulation, intravitreal anti-vascular endothelial growth factor drugs, in travitreal steroids, and pars plana vitrectomy. The Ozurdex implant was approved by the Food and Drug Administration in 2009 for the treatment of these conditions, but remains contraindicated in patients with ocular or periocular infections, advanced glaucoma, or a compromised posterior lens capsule^(7)^.

Crystalline lens injuries during the implant injection procedure have been previously reported, with an incidence of 0.009%. In contrast to regular intravitreal injection, Ozurdex uses a 22-gauge delivery system that propels the copolymer pellet with high speed into the vitreous cavity. The risk of an inadvertent lens injury increases if the physician uses an improper injection technique, is inexperienced or in training, and/or if the patient’s head is suddenly moved during the injection^(8)^. In this case, an improper technique, which caused anterior movement of the Ozurdex delivery system during injection, resulted in the crystalline lens injury. In previous reports of similar complications, immediate surgical intervention has been suggested if the lens injury leads to significant cataract formation, sudden IOP increase, and/or corneal decompensation^(9)^.

In this case, we followed a more conservative approach because IOP was stable, specular microscopy findings were normal, and no immediate cataract formation was present; these characteristics were similar to those of the patient described by Baskan et al.^(10)^.

Crystalline lens injury due to intravitreal Ozurdex is a rare complication that is typically attributed to the injection procedure. An immediate surgical or conservative approach should be considered on an individual basis. Factors to consider in such cases include the following: increased IOP, cataract formation, corneal endothelium toxicity, and worsening of CME. Although the situation could be resolved by phacoemulsification with implantation of a sulcus IOL, the surgeon should be prepared to perform pars plana vitrectomy if surgical complications arise.
